# Upper limb artery segmental occlusions due to chronic use of ergotamine combined with itraconazole, treated by thrombolysis

**DOI:** 10.1186/1477-9560-9-13

**Published:** 2011-08-30

**Authors:** Edoardo Cervi, Stefano Bonardelli, Giuseppe Battaglia, Federico Gheza, Roberto Maffeis, Franco Nodari, Roberto Maroldi, Stefano M Giulini

**Affiliations:** 1 Surgical Clinic, University of Brescia, 25123, Brescia, Italy; 2 Radiologic clinic, University of Brescia, 25123, Brescia, Italy

**Keywords:** Ergotamine, fibrinolysis, upper limb ischemia, Itraconazole

## Abstract

**Background:**

The ergotamine tartrate associated with certain categories of drugs can lead to critical ischemia of the extremities. Discontinuation of taking ergotamine is usually sufficient for the total regression of ischemia, but in some cases it could be necessary thrombolytic and anticoagulant therapy to avoid amputation.

**Case report:**

A woman of 62 years presented with a severe pain left forearm appeared 10 days ago, with a worsening trend. The same symptoms appeared after 5 days also in the right forearm. Physical examination showed the right arm slightly hypothermic, with radial reduced pulse in presence of reduced sensitivity. The left arm was frankly hypothermic, pulse less on radial and with an ulnar humeral reduced pulse, associated to a decreased sensitivity and motility.

Clinical history shows a chronic headache for which the patient took a daily basis for years Cafergot suppository (equivalent to 3.2 mg of ergotamine).

From about ten days had begun therapy with itraconazole for vaginal candidiasis. The Color-Doppler ultrasound shown arterial thrombosis of the upper limbs (humeral and radial bilateral), with minimal residual flow to the right and no signal on the humeral and radial left artery.

**Results:**

Angiography revealed progressive reduction in size of the axillary artery and right humeral artery stenosis with right segmental occlusions and multiple hypertrophic collateral circulations at the elbow joint. At the level of the right forearm was recognizable only the radial artery, decreased in size. Does not recognize the ulnar, interosseous artery was thin. To the left showed progressive reduction in size of the distal subclavian and humeral artery, determined by multiple segmental steno-occlusion with collateral vessels serving only a thin hypotrophic interosseous artery.

Arteriographic findings were compatible with systemic drug-induced disease. The immediate implementation of thrombolysis, continued for 26 hours, with heparin in continuous intravenous infusion and subsequent anticoagulant therapy allowed the gradual disappearance of the symptoms with the reappearance of peripheral pulses.

**Conclusion:**

Angiography showed regression of vasospasm and the resumption of flow in distal vessels. The patient had regained sensitivity and motility in the upper limbs and bilaterally radial and ulnar were present.

## Case report

A 62 years woman was admitted in our Institute complaining a strong bilateral forearm pain. The pain left upper limb had appeared 10 days ago and after 5 days was also presented on the right side.

The history revealed that the patient was suffering from migraine with aura and therefore she took Cafergot^® ^(ergotamine) for 5-6 years, 2-4 mg/day. The patient, with these doses of ergotamine, could control the headaches that immediately recurred in case of drug withdrawal.

Moreover, 10 days before she had started taking 4 tablets per day of Sporanox^® ^(itraconazole) for a vaginal candidiasis.

The pain was first discontinuous, associated with effort, and only after become continuous. Physical examination showed the right arm hypothermic, with reduced radial pulse, in the absence of motility deficiency and loss of sensation.

The left arm was frankly hypothermic in the absence of radial and ulnar humeral loss of pulse.

At the CW-Doppler control: absence of signal in the left radial and ulnar artery and demodulated signal at humeral artery; on the right, demodulated signal at the radial and ulnar arteries.

After a Color-Doppler ultrasound of the upper limbs, which showed bilateral humerus thrombosis with minimal residual flow on the right radial artery and no signal on the left radial artery, the patient was subjected to emergency upper limb angiography for possible thrombolytic therapy.

**Figure 1:**
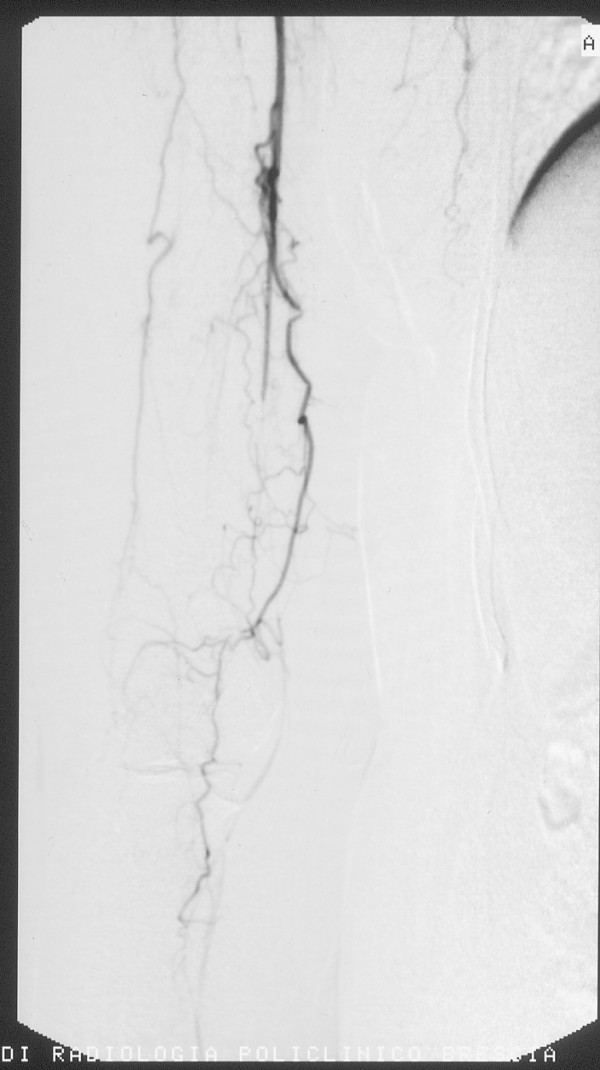
**First angiography, showing the right arm with only the radial artery, decreased in size**.

Under local anesthesia, a right femoral approach was performed through a 4F introducer sheath. Angiography revealed a progressive reduction of caliber at the right axillary artery with a steno-occlusions of the right humeral artery and multiple segmental hypertrophic collateral circulation in the elbow joint. At the level of the right forearm was recognizable only the radial artery, decreased in size. To the left, showed progressive reduction in size of the distal subclavian and humeral arteries, determined by multiple segmental steno-occlusion with collateral hypotrophic vessels that belatedly revascularized only a thin interosseous artery (Figure 1).

The arteriographic findings were compatible with systemic drug induced disease: ergotamine therapy was suddently suspended.

The thrombolytic treatment was performed through a 4F catheter with the tip placed at the origin of the brachial artery: after a 60.000 urokinase U.I injection, a slow intra-arterial infusion of urokinase and heparin during the following hours was performed.

At the end of the procedure, after the administration of about 1.300.000 U.I of urokinase, patency of the brachial and ulnar artery was re-established, obtaining a good perfusion of the hand; however, a concentric stenosis was still detectable in the distal brachial artery and in the ulnar and radial arteries. The immediate implementation of thrombolysis, continued for 26 hours, keeping the patient anticoagulation with heparin infusion pump (15.000 IU/24 h, to maintain a PTT of 50-55 sec) led to the gradual disappearance of the symptoms of patients with recurrence of the peripheral pulses.

Serial angiograms during the procedure shew a gradual thrombolytic recanalization of the artery occluded, in the presence of angiographic signs of concentric wall thickening of vasculitic nature.

**Figure 2:**
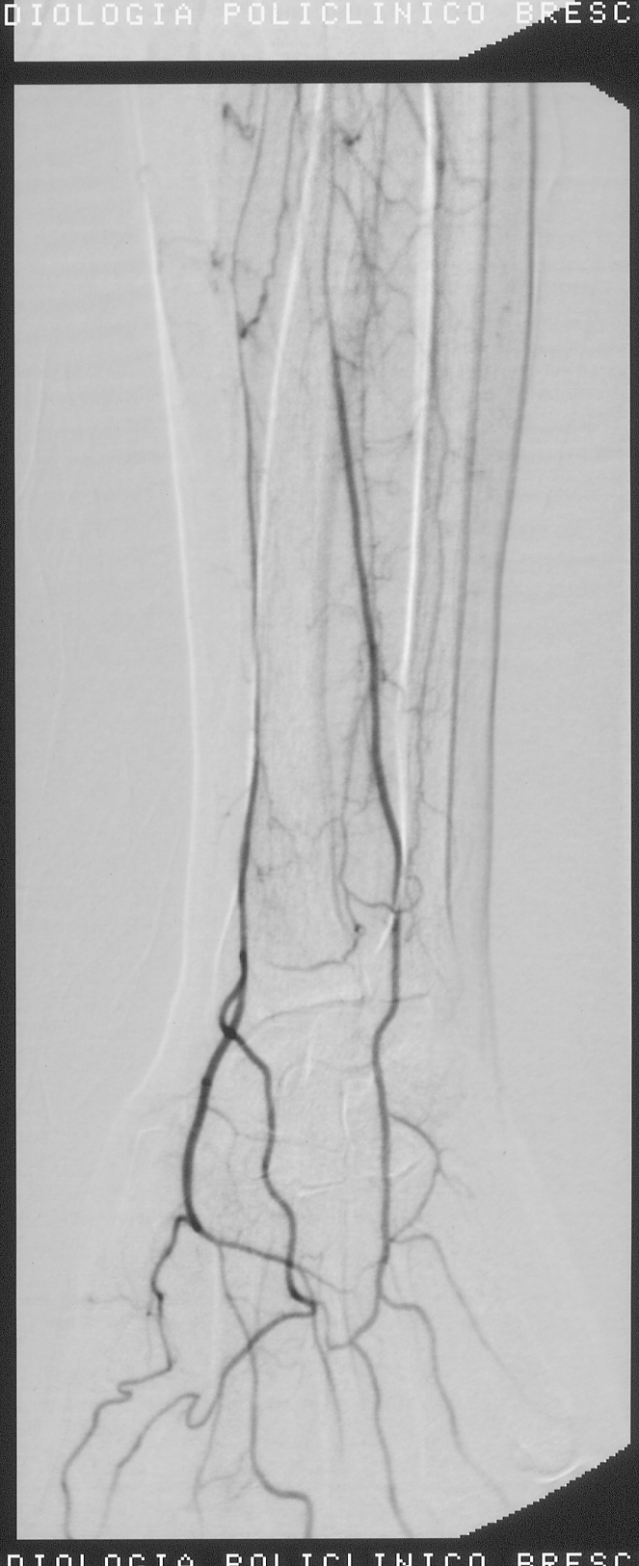
**Last angiography, after thrombolysis, with a normal vascularisation of the right forearm**.

The final angiographic control shew regression of vasospasm and the resumption of flow in distal vessels (Figure 2).

Treatment with heparin during hospitalization was imbricate with oral anticoagulant therapy (Coumadin^®^, maintaining an INR between 2.0 and 3.0), and supported for 12 days with intravenous prostaglandin treatment (Prostavasin^® ^20 mg/day).

To complete diagnosis, were sought: anticardiolipin, anti-beta 2 GPIa IgG IgM to exclude immunological genesis (all doses appeared to be normal); genetic testing (factors II and V Leiden) and homocysteine assay for exclude metabolic diseases with genetic etiology (all doses appeared to be normal); aortic arch arteriography allowed to exclude a primary disease like Takayasu arthritis type. Moreover, we proposed to test antithrombin, protein C/S deficiency and the prothrombin mutation (20210A) and all results were negative.

During hospitalization, three days after ergotamine discontinuation, the patient presented a strong trend to worsening headache accompanied by nausea and vomiting that required an implemented treatment with benzodiazepines (10 drops of Valium three times daily), inhibitors of re-uptake of serotonin (Citalopram 10 mg daily) and an associated steroid (Bentelan) in case of resistance.

The patient was discharged completely asymptomatic after two weeks of hospitalization. At two weeks, three and six months the patient was in subjective well-being, in absence of motility and sensitivity deficiency. Radial and ulnar pulses were present bilaterally and a Color-Doppler ultrasound showed a normal blood supply of the upper limbs. Even the patient's headache was controlled by the new implemented therapy after ergot discontinuation.

## Discussion

The Ergotamine is an alkaloid produced by a fungus, Claviceps purpurea [1], described in the history of epidemics caused by ingestion of food contaminated with this fungus [2] and subsequently used to treat migraine, for its activation of the sympathetic system [3,4].

Ergotamine induced ischemia is rare but potentially serious complications may lead through two mechanisms: vasospasm and thrombosis. Toxicity arises from chronic therapeutic doses, acute ingestion of excessive doses of the drug and acute ingestion of therapeutic doses in patients with hypersensitivity.

The literature shows a surprisingly low number of clinical cases using angiographic evidence of arterial stenosis showed a rapidly reversible after discontinuation of ergotamine [5-7].

McKiernan et al reported a case of severe vasospasm of the lower extremities (and renal artery) induced by ergotamine tartrate [8].

Martinet et al. used the Duplex ultrasound showing multiple areas of vasospasm in the lower limbs in a woman of 38 years who suffered from "chronic migraine" treated with ergotamine tartrate and resolved after infusion of sodium nitroprusside and epidural analgesia [9].

D'Amore et al. showed a severe arterial stenosis to load the lower extremities in a patient taking Cafergot for migraine headaches [10].

Regarding the treatment of limb ischemia upper and lower drug discontinuation is certainly the first procedure to be implemented, but not always sufficient to reverse the disease in place.

In severe forms of ischemia refractory to therapy surgical sympathectomy with balloon dilatation or arterial dilation can be effective, however the patient management depends on clinical and instrumental monitoring of vasospasm, especially in cases of advanced ischemia.

Bongard and Bounameaux infused sodium nitroprusside in 6 patients with severe acute lower limb ischemia due to ergotamine tartrate or dihydroergotamine recruitment, the vasospasm was relieved within 48 hours in all patients except one [11].

Thrombolysis was effective in the patient described by D'Amore et al. [10]. In refractory cases, the PTA has been proposed.

The ergotamine discontinuation with the infusion of nitroprusside or nitroglycerin appears to solve the problem in most cases [12].

In our case the patient took ergotamine chronically for about three years without any reported problems, the addition of an antifungal (itraconazole) has caused the artery vasospasm and thrombosis of the upper limbs. The interaction of erythromycin and ergotamine at low doses can cause ischemia of the lower extremities [13]. Similarly, taking 3 mg ergotamine over 5 days led to a severe vasospasm requiring amputation transmetatarsale when it was administered to a patient treated with the drug anti-HIV ritonavir [14]. Recently, Baldwin et al. described a case of ergotamine toxicity in an HIV-positive patients treated with protease inhibitors [15].

In our case, we decided to start thrombolytic therapy continued for 26 hours maintaining the patient on anticoagulation with heparin infusion (maintaining a PTT of 50-55 sec) for 3 days, then overlapped with an oral anticoagulant therapy. At the same time a vasoactive therapy was also carried out for 15 days. This attitude has led to the gradual resolution of the ischemic and the clinical point of view with the reappearance of peripheral pulses, both in terms of radiological with complete resolution of vasospasm and thrombotic process. In conclusion in the literature has not documented any cases of ischemia with the combination of ergotamine and itraconazole, should we believe an aggressive therapeutic approach in cases of significant clinical impairment and instrumental. We believe that in cases of vasospasm associated with arterial thrombosis thrombolytic treatment, when necessary, PTA and vaso-active therapy provides an excellent immediate result avoiding prolonged ischemic pain related complications.

## Consent

Written informed consent was obtained from the patient for publication of this case report and accompanying images. A copy of the written consent is available for review by the Editor-in-Chief of this journal.

## Competing interests

The authors declare that they have no competing interests.

## Authors' contributions

EC, GB and SB had the primary role in patient care, EC and FG wrote the manuscript, FG made the literature research, SB, SMG and RM revised the manuscript. All authors read and approved the final version.
